# Combined Management of a Rare Case of an Incarcerated Intrauterine Device: Case Report and Review of the Literature

**DOI:** 10.1155/crog/7806325

**Published:** 2026-03-09

**Authors:** Stefano Restaino, Federico Paparcura, Elisa Rizzante, Silvia Zermano, Annalisa Graziano, Antonio Ferraro, Margherita Cuman, Alice Poli, Martina Arcieri, Lorenza Driul, Giuseppe Vizzielli

**Affiliations:** ^1^ Clinic of Obstetrics and Gynecology, “S. Maria della Misericordia” University Hospital, Azienda Sanitaria Universitaria Friuli Centrale (ASUFC), Udine, Italy; ^2^ Biomedical Sciences, Gender Medicine, Child and Women Health, University of Sassari, Sassari, Italy, uniss.it; ^3^ Department of Medicine (DMED), University of Udine, Udine, Italy, uniud.it

**Keywords:** case report, hysteroscopy, intrauterine system (IUS), laparoscopy, uterine embedment

## Abstract

**Background:**

Hormonal intrauterine systems are widely used for long‐acting reversible contraception and for managing gynecological conditions such as heavy menstrual bleeding and endometrial hyperplasia. Although rare, complications such as uterine embedment and perforation can occur, potentially leading to device malfunction or injury displacement. This is the first reported case describing the removal of a fully incarcerated hormonal intrauterine system using a combined hysteroscopic and laparoscopic approach. Our findings suggest that this technique may offer a safe and effective alternative for managing cases of embedded intrauterine devices where conventional hysteroscopic removal is unsuccessful.

**Case Presentation:**

We report the case of a 35‐year‐old asymptomatic woman with a hormonal intrauterine system fully embedded in the posterior myometrial wall, positioned 4.3 mm from the uterine serosa. The device was initially confirmed to be intracavitary upon insertion but was later found to have migrated into the myometrium during a routine 30‐day follow‐up ultrasound. Initial attempts at hysteroscopic removal failed due to poor visualization and a broken retrieval string. Given the high risk of iatrogenic injury, a combined laparoscopic–hysteroscopic approach was planned. Under laparoscopic visualization and transrectal ultrasound guidance, a careful myometrial excision using a hysteroscopic‐angled loop was performed, successfully freeing and removing the intrauterine device. A bilateral salpingectomy was also conducted at the patient′s request.

**Conclusion:**

A combined laparoscopic–hysteroscopic approach under ultrasound guidance may be regarded as a viable technique for complex cases of intrauterine device incarceration within the myometrium, minimizing the risk of uterine perforation and adjacent organ injury.

## 1. Introduction

Hormonal intrauterine systems (IUSs) rank among the most commonly used forms of long‐acting reversible contraception due to their high efficacy and safety [1]. In recent years, IUSs have been increasingly utilized not only for contraception but also for treating conditions such as heavy menstrual bleeding and endometrial hyperplasia [2].

Complications associated with IUSs include failed insertion, pain, vasovagal reactions, infections, menstrual abnormalities, pregnancy with an IUS in situ, expulsion, and nonvisible strings [2–4]. Although rare, complications such as uterine incorporation (embedding of the IUS in the myometrium) and uterine perforation (penetration beyond the uterine serosa) occur in approximately 1 in 1000 insertions [5, 6]. The main risk factors for perforation include breastfeeding and insertion within the first 6 weeks postpartum [2, 6, 7].

Symptoms of uterine embedment and/or perforation vary, ranging from asymptomatic cases to severe abdominal pain and abnormal vaginal bleeding [8–10]. In rare instances, distant migration of the device may occur, potentially causing injury to intra‐abdominal structures [11]. Uterine perforation is often asymptomatic unless severe complications arise, such as active bleeding, bowel or bladder perforation, fistula formation, or sepsis [10, 12].

The preferred surgical approach for displaced IUSs depends on their location. In cases of intra‐abdominal dislocation, laparoscopy is the recommended surgical method for device removal [11]. Conversely, for IUSs displaced within the uterus, hysteroscopy is the first‐line technique and can also be performed on an outpatient basis. Using the hysteroscope, the device can typically be visualized and removed by following the retrieval strings [13].

We described the case of an asymptomatic woman with IUS migration into the myometrium and provided a systematic review of the literature.

## 2. Case Report

A 35‐year‐old woman with two previous pregnancies and a medical history of obesity (BMI: 38) presented to our clinic after a positive home pregnancy test. Transvaginal ultrasound confirmed a pregnancy at 10 weeks of gestation. The patient requested a voluntary termination of her pregnancy via a surgical procedure, combined with the insertion of an IUS.

**Figure 1:**
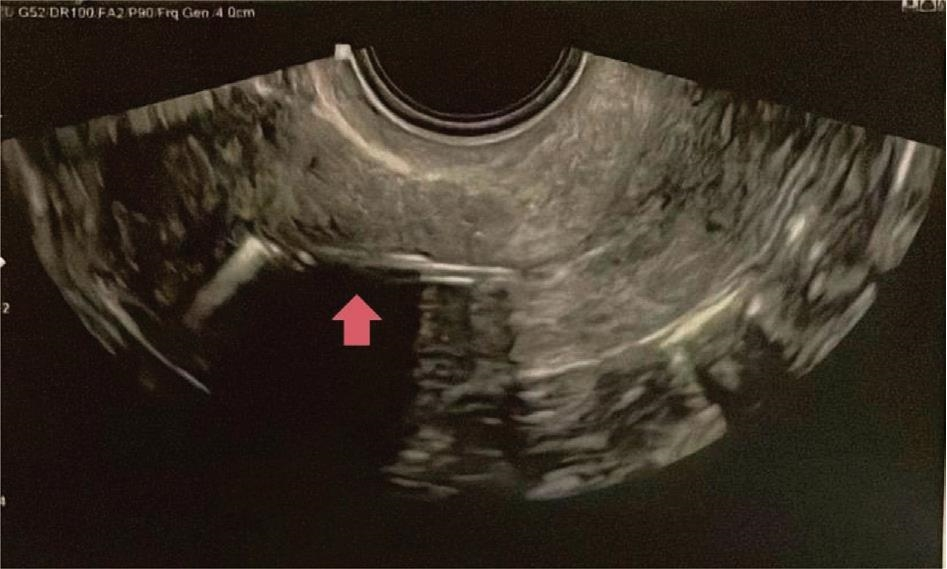
Postinsertion ultrasound image of the IUS. The device is correctly positioned.

Following hysterosuction using the Karman method and in accordance with hospital protocol, a 13.5‐mg levonorgestrel‐releasing IUS was inserted at the end of the procedure. Postprocedure ultrasound confirmed the correct positioning of the intrauterine device (Figure 1).

**Figure 2:**
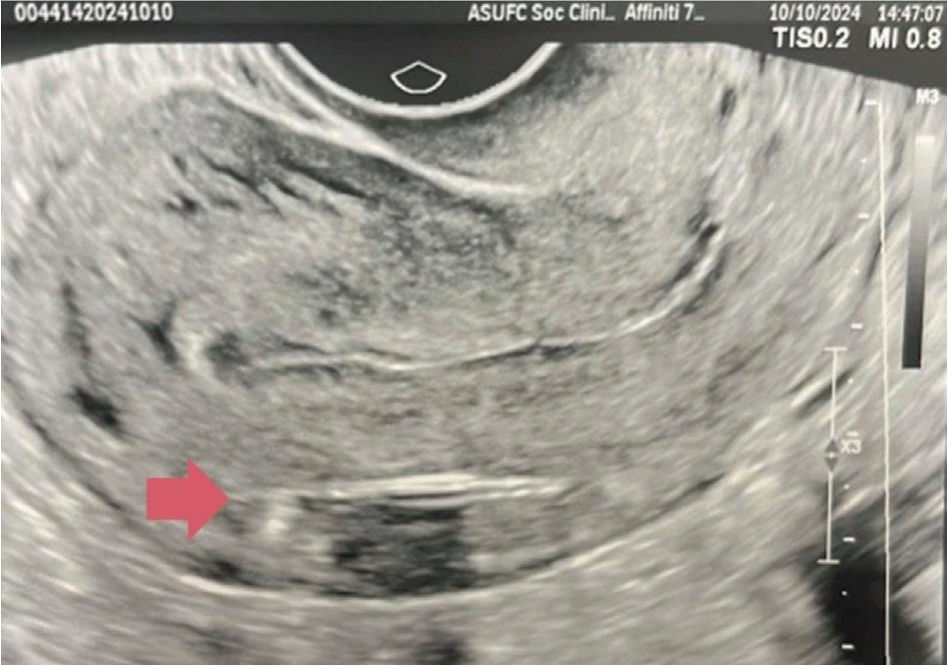
IUS embedded in the myometrium at the 30‐day follow‐up.

At the routine 30‐day follow‐up, transvaginal ultrasound revealed that the IUS had migrated into the myometrium (Figure 2). However, the patient remained asymptomatic and reported no fever or abdominal pain. Attempts to remove the device by gently pulling the retrieval strings were unsuccessful. To minimize the risk of device rupture and considering its position, a hysteroscopic removal attempt was scheduled using the Bettocchi technique.

During preadmission, a 3D ultrasound confirmed that the IUS was fully embedded within the posterior myometrial wall, 4.3 mm from the uterine serosa. During diagnostic hysteroscopy, the endometrium was inadequately prepared, resulting in poor visibility despite the procedure being performed under ultrasound guidance. Additionally, the IUS‐retrieval string broke during attempts at removal. To minimize the risk of perforation and enhance visibility, the procedure was postponed, and endometrial preparation was initiated with norethisterone acetate (10 mg daily).

The patient was presented with various therapeutic strategies: a combined laparoscopic–hysteroscopic approach; conservative management, leaving the displaced IUS in the uterine wall; an abdominal approach (laparoscopic/laparotomic) with total hysterectomy in case of hysteroscopic failure. After thorough counseling, the patient opted for laparoscopically assisted hysteroscopic removal.

Three months after IUS insertion, the patient underwent laparoscopically‐assisted operative hysteroscopy. After introducing the umbilical optical trocar and inducing pneumoperitoneum, two additional 5‐mm accessory trocars were placed in the left and right iliac fossae. Abdominal exploration revealed a uterus of normal morphology and volume, with no visible signs of the IUS through the serosa.

**Figure 3:**
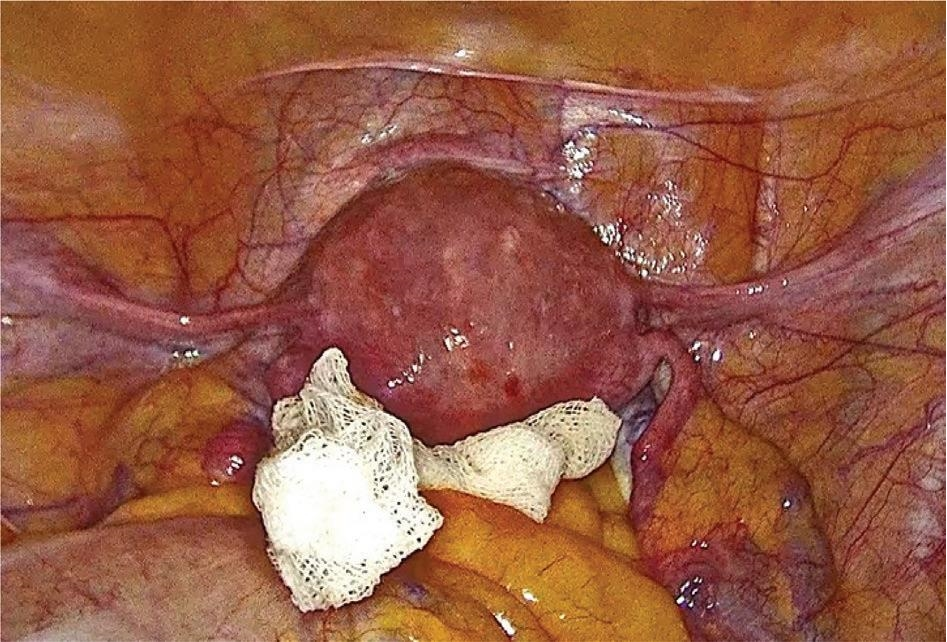
Laparoscopic view of the uterus. A sponge was placed in the pouch of Douglas to reduce the risk of iatrogenic complications.

To reduce the risk of iatrogenic injury to the sigmoid colon, a sponge was placed in the Douglas pouch to displace the posterior uterine wall (Figure 3). Diagnostic hysteroscopy using the Bettocchi technique revealed a regular endometrium consistent with progestogen stimulation, with no evidence of the IUS or its marker. Given the clinical scenario, we proceeded with operative hysteroscopy using a mini‐resectoscope. Under transrectal ultrasound guidance and laparoscopic visualization, a careful myometrial excision was performed using a hysteroscopic‐angled loop until the incarcerated IUS was visualized. The device was fully freed and removed using an equatorial loop. The video of the procedure is available as supporting material (Video S1).

Given the patient′s medical history and after appropriate counseling, she requested a bilateral salpingectomy, which was performed at the end of the hysteroscopic procedure.

**Figure 4:**
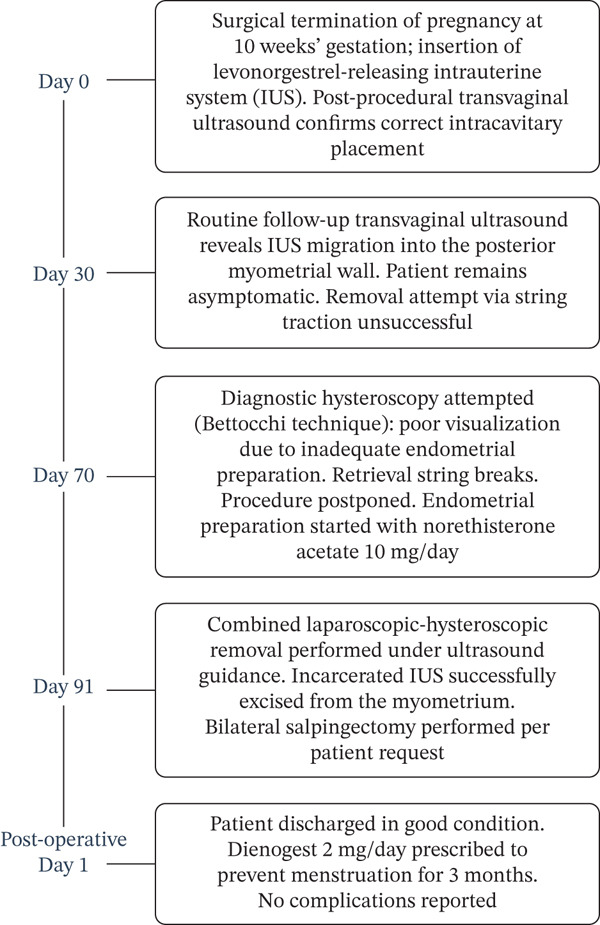
Case report timeline.

The patient was discharged the day after surgery. To prevent the appearance of menstruation, she was prescribed dienogest (2 mg per day) for 3 months. No intraoperative or postoperative complications occurred. Figure 4 summarizes the timeline of the patient′s clinical history.

Written informed consent was obtained from the patient for the publication of this case report and any accompanying images.

## 3. Discussion and Review of the Literature

This is the first reported case describing the removal of a fully incarcerated IUS within the myometrium, without retrieval strings, using a combined laparoscopic–hysteroscopic surgical technique. Intrauterine device insertion can lead to various complications, including failed placement, pain, vasovagal reactions, infections, menstrual abnormalities, and device expulsion [3, 4]. Although uterine embedment and perforation are rare, they should be considered, especially due to the increased use of IUSs in recent decades [12]. The precise mechanism underlying IUS perforations remains unclear, and several etiological theories have been proposed. One theory suggests that complete perforation occurs during device insertion, causing the IUS to be released beyond the serosa. Another theory states that the IUS is initially placed correctly but later undergoes transmural migration, resulting in perforation [14]. According to Goldstuck et al., the combination of forces exerted during IUS insertion and those generated by the myometrium plays a fundamental role in uterine embedment and perforation [15].

Some cases of IUS dislocation and its management have been described in the literature (Table 1). In these case reports, the IUS was displaced into the pelvic and abdominal cavity, attaching to structures such as the uterus, rectum, bladder, bowel, broad ligament, and omentum [16–18]. Management in these cases involved imaging to determine the device′s location, followed by accurate treatment. If parts of the IUS retrieval strings were visible on a 2D transvaginal ultrasound, a 3D ultrasound was subsequently performed to better evaluate its relationship with the endometrium and myometrium [19]. If the IUS was not found in the endometrial cavity, a computed tomography scan was performed for enhanced diagnostic accuracy and precise localization [20, 21]. According to the World Health Organization′s recommendations, displaced IUSs should be removed due to the risk of bowel injury, chronic pelvic pain, and infertility. Consequently, in such cases, IUS removal was performed via laparoscopic surgery [16, 22].

**Table 1:** Studies review.

Authors	Title	Type	*N*° cases	Location	Removal
Restaino S. et al.	Discovery of a Missing Intrauterine System in the Peritoneal Cavity During Cervical Cancer Surgery: A Case Report	Case report	1	Base of the mesentery at the caecum level	LPT
Markovitch O. et al.	Extrauterine Mislocated IUD: Is Surgical Removal Mandatory?	Case report	3	1 = lying on the rectosigmoid1 = sigmoid epiploic fat posterior to the uterus1 = lying on the mesentery of the rectum	LPS
Vilos G.A. et al.	Algorithm for Nonvisible Strings of Levonorgestrel Intrauterine System	Case report	1	Right side of the pelvis, adhered to the omentum	LPS
Onalan G. et al.	Extrauterine Displaced Intrauterine Devices: When Should They Be Surgically Removed?	Case report	1	Within a pelvic abscess in the Douglas pouch	LPS
Mahmoud M.S. et al.	Computed Tomography‐Assisted Laparoscopic Removal of Intraabdominally Migrated Levonorgestrel‐Releasing Intrauterine Systems	Case report	3	1 = left paracolic gutter, partially adherent and embedded to the omental fat1 = anterior to the right ovary1 = inferior to the coecum, completely embedded in the omentum	LPS
Balci O. et al.	Removal of Intra‐Abdominal Mislocated Intrauterine Devices by Laparoscopy	Retrospective study	15	6 = Douglas3 = posterior uterine wall3 = in the adnexa2 = in the omentum1 = embedded in the rectal serosa	LPS
Inal H.A. et al.	Successful Conservative Management of a Dislocated IUD	Case report	1	Right lower front part of the abdomen	Conservative management
Mosley F.R. et al.	Elective Surgical Removal of Migrated Intrauterine Contraceptive Devices From Within the Peritoneal Cavity: A Comparison Between Open and Laparoscopic Removal	Systematic review	129	42 = free in pelvis9 = attached to uterus2 = tubo‐ovarian3 = attached to rectum1 = attached to bladder5 = attached to broad ligament41 = embedded in omentum6 = free in peritoneal cavity13 = attached to bowel4 = mass of bowel and pelvic structures3 = mass of omentum and pelvic structures	93 = LPS27 = LPS/LPT9 = LPT
Ferguson C.A. et al.	Transmural Migration and Perforation of a Levonorgestrel Intrauterine System: A Case Report and Review of the Literature	Case report	1	Migrated further out of the uterus with only the stem still intrauterine	LPS
Soleymani Majd H. et al.	Migration of Levonorgestrel IUS in a patient With Complex Medical Problems: What Should Be Done?	Case report	1	In the anteriorabdominal wall, just above the caecum	Conservative management (patient declared unfit for surgery)

In the literature, only the case report by Inal et al. highlights the success of the conservative approach in managing an IUD located in the abdominal cavity. The patient underwent a 3‐year follow‐up without experiencing any complications or symptoms [23].

Our case, a unique instance in literature, underscores the importance of careful surgical planning and the need for direct uterine control. At the time of diagnosis, the IUS was completely embedded within the myometrial thickness, making it invisible by hysteroscopy. Furthermore, the retrieval strings were absent at the time of removal, having been removed in a previous attempt, thereby increasing the extraction difficulty. Our case also marks the first reported instance of a combined hysteroscopic and laparoscopic approach for IUS removal. After an unsuccessful attempt at removal via hysteroscopy using the Bettocchi technique, a novel strategy was devised. This involved operative hysteroscopy using a mini‐resectoscope in combination with laparoscopy. The decision to include a laparoscopic approach was based on two considerations: the need for direct uterine control to prevent perforation and potential damage to surrounding organs and the patient′s request for salpingectomy to prevent future pregnancies. Additionally, to ensure precise guidance of the resectoscope loop and accurate positioning of the IUS, the hysteroscopy was performed under transrectal ultrasound guidance. Therefore, this combined hysteroscopic and laparoscopic approach under transrectal ultrasound guidance could potentially be used in the future as a treatment for IUS incarcerated within the myometrium, as in the case of our patient.

## Funding

This study was supported by Universita degli Studi di Sassari as part of the Wiley—CRUI‐CARE agreement, which facilitated open access publishing.

## Consent

Patient consent was obtained.

## Conflicts of Interest

The authors declare no conflicts of interest.

## Supporting information


**Supporting Information** Additional supporting information can be found online in the Supporting Information section. Video S1: Hysteroscopic removal of embedded IUS.

## Data Availability

The data that support the findings of this study are available on request from the corresponding author. The data are not publicly available due to privacy or ethical restrictions.
